# TDP-43 is coming to the cottage: A new tool to study neurodegenerative diseases

**DOI:** 10.1371/journal.pbio.3003697

**Published:** 2026-03-25

**Authors:** Stephanie L. Rayner, Aaron D. Gitler

**Affiliations:** 1 Department of Genetics, Stanford University School of Medicine, Stanford, California, United States of America; 2 Motor Neuron Disease Research Centre, Macquarie Medical School, Faculty of Medicine, Health, and Human Sciences, Macquarie University, Sydney, New South Wales, Australia

## Abstract

A common hallmark of many neurodegenerative diseases is the mislocalization and aggregation of proteins in the brain, but modeling these key pathological events has been challenging. This Primer explores a recent study in PLOS Biology that develops a new cell-based method to monitor TDP-43 aggregation in real time and test therapeutic interventions.

## Teaser

In some neurodegenerative diseases, the protein TDP-43 is both lost from the nucleus and forms clumps in the cytoplasm. These two pathologies can be challenging to model, but a study in *PLOS Biology* presents a new system that captures both features.

Nearly all neurodegenerative diseases share a common feature—the accumulation of protein clumps in the brain [1]. One promising therapeutic avenue has been trying to target and dispose of the protein aggregates. This has been empowered by early studies in cell and animal models that mimic pathological cascades seen in disease. But for two neurodegenerative diseases, amyotrophic lateral sclerosis (ALS, also known as motor neuron disease) and frontotemporal dementia (FTD), things are a bit more complicated. The protein culprit in nearly all ALS cases and about half of FTD cases is an RNA-binding protein called TDP-43 [2]. It is not going to be as simple as designing a drug to target and remove TDP-43, because in addition to building up in the cytoplasm it also causes trouble when it is lost from the nucleus and cannot carry out its normal function as a regulator of gene expression. Simultaneously modeling both hallmarks has been challenging, but a new paper by Mamede and colleagues published in this issue of *PLOS Biology* [3] presents a cell-based system that captures both cytoplasmic aggregation and resulting loss of TDP-43 nuclear function.

Normally, TDP-43 is localized in the nucleus where it functions to ensure correct messenger RNA processing. When TDP-43 is depleted from the nucleus, it leads to the disruption of transcript processing, the emergence of aberrant RNAs, and loss of important neuronal proteins [4]. And on top of that, there seems to be a self-perpetuating cycle where TDP-43 regulates its own expression, by binding to its RNA transcript and controlling the forms of TDP-43 transcripts and protein that are generated by the cell [5]. Researchers have attempted to model TDP-43 pathology using various approaches ranging from animal models to neuron cultures [6]. Some teams focused on overexpressing TDP-43 at high levels to mimic the cytoplasmic aggregation component. Other teams used gene silencing approaches to reduce levels of TDP-43 to mimic the loss-of-function component. Others mutated TDP-43’s nuclear localization signal to keep it out of the nucleus, causing it to aggregate in the cytoplasm. All these approaches have been informative but unfortunately none have been able to capture all the disease-relevant features. The Holy Grail for the field has been to generate a model that exhibits cytoplasmic aggregation, loss from the nucleus, and disrupted autoregulation.

**Fig 1:**
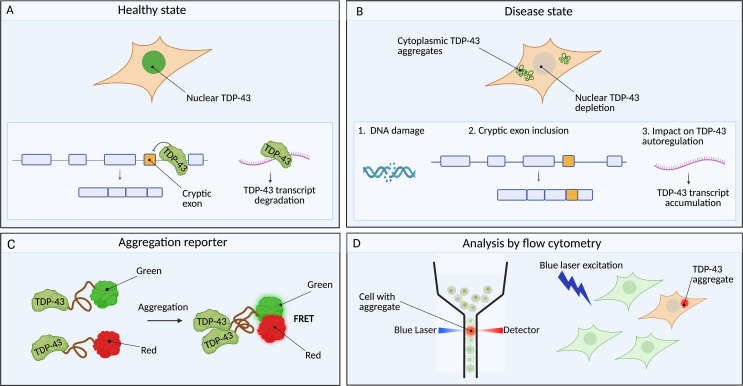
A cell-based reporter of TDP-43 disease states. **(A)** In healthy cells, TDP-43 is predominantly nuclear. Within the nucleus, TDP-43 plays important roles in repressing the inclusion of cryptic exons as well as regulating its own transcript levels and isoforms. This ensures appropriate levels of functional TDP-43 within the cell. **(B)** In disease, TDP-43 aggregates in the cytoplasm and is lost from the nucleus, leading to both gain-of-function and a loss-of-function. Loss of nuclear function leads to multiple detrimental events. These include DNA damage as well as the inclusion of cryptic exons in transcripts (depicted as orange boxes in the diagram), in turn leading to the production of incorrectly processed transcripts. In addition, disrupted TDP-43 autoregulation leads to unchecked accumulation of the protein’s own transcript. **(C)** The reporter line developed by Mamede and colleagues enables the study of TDP-43 aggregation in response to stimuli—in this case exposure to TDP-43 protein aggregates that originated from patients with FTD. The reporters are composed of the C-terminal domain of TDP-43 fused to either a green or red fluorescent protein (FRET pairs). When the reporter aggregates, the proximity of TDP-43 FRET pairs enables the quantitative evaluation of aggregation within each cell. **(D)** Protein aggregates can be quantified after excitation using a blue laser. Cells containing TDP-43 aggregates produce a FRET-positive signal that can be measured using flow cytometry whilst cells that do not have aggregates do not produce a signal. Image created in BioRender. Cheng, F. (2026) https://BioRender.com/9wmr4eb.

To get started, Mamede and colleagues built a cell-based system to allow them to monitor if and when TDP-43 starts aggregating (Fig 1). They cleverly used a physics principle called Förster Resonance Energy Transfer (FRET), which occurs when two molecules come within a certain distance of each other—a “donor” molecule emits energy and an “acceptor” molecule gets excited and fluoresces. Importantly, when this energy is transferred the donor molecule fluorescence diminishes. They engineered their cells to express a part of TDP-43 hooked up to one of two different fluorescent proteins—a green one (mClover) or a red one (mRuby). When TDP-43 starts aggregating, the mClover and mRuby fusion proteins come into close proximity and FRET occurs. In this way, they could collect their cells and put them into a flow cytometer, which shines a laser to excite the green fluorescent protein and has a sensitive detector to find and measure the ones with TDP-43 aggregates that emit red fluorescence. And this is highly quantitative—the more TDP-43 aggregates the brighter the cells become.

To initiate aggregation, the authors extracted TDP-43 seeds from the brains of individuals who had lived with FTD. They then added these seeds to their cells. The seeds rapidly entered the cells and caused FRET-induced fluorescence—meaning TDP-43 was aggregating. The engineered TDP-43 fragment was not the only thing that aggregated—the endogenous TDP-43 also aggregated and the authors found that it even harbored some of the telltale signs seen in disease like phosphorylation and recruitment of the ubiquitin-binding protein 62 (p62). In their system, TDP-43 aggregation was followed by the gradual depletion of nuclear TDP-43, leading to subsequent loss-of-function events like impaired RNA processing and DNA damage. Further, they showed that TDP-43 aggregation disrupts its own autoregulation mechanism—exposure to disease-derived TDP-43 seeds led to the upregulation of TDP-43’s own transcript levels. Thus, the authors have built a system that in one fell swoop recapitulates all three fundamental facets of TDP-43 pathology seen in disease. And in so doing, they reveal a cascade of events starting when tiny TDP-43 clusters enter healthy cells, corrupt normal TDP-43 proteins to coax them to aggregate, and then this culminates in TDP-43 getting dragged out of the nucleus and its RNA targets becoming dysregulated.

This new cell model offers many exciting opportunities for the field to study what makes TDP-43 aggregate and to test therapies aimed at mitigating it. As proof of concept, they tested another protein called Ataxin-2, which had previously been shown to facilitate TDP-43 aggregation [7]. Reducing Ataxin-2 levels resulted in decreased TDP-43 aggregation and, excitingly, restored the normal RNA processing events, indicating that they had corrected both TDP-43 aggregation and loss-of-function phenotypes.

A couple of limitations to this model need mentioning. The authors’ cell model generates TDP-43 aggregates, but higher resolution structural studies will be important to determine if and how these assemblies resemble ones seen in human brain [8]. Also, the authors need to express their TDP-43 reporter system at high levels to clearly see the initial gain-of-function events, which are not clearly seen in wild-type cells seeded with FTD aggregates. This suggests overexpression of this construct is necessary to see both events clearly.

This paper not only gives us a novel TDP-43 biosensor which we can use to study both gain-of-function and loss-of-function events, it also teaches us that these hallmarks are linked. These tools and similar ones generated by others [9,10] now provide the scientific community with a means to study key features of TDP-43-linked diseases and develop strategies to manage them.

## Abbreviations

ALS: Amyotrophic Lateral Sclerosis
FRET: Förster Resonance Energy Transfer
FTD: Frontotemporal Dementia
